# Female reproductive tract-organ axes

**DOI:** 10.3389/fimmu.2023.1110001

**Published:** 2023-01-31

**Authors:** Kazuhide Takada, Vyacheslav G. Melnikov, Ryoki Kobayashi, Shihoko Komine-Aizawa, Noriko M. Tsuji, Satoshi Hayakawa

**Affiliations:** ^1^ Division of Microbiology, Department of Pathology and Microbiology, Nihon University School of Medicine, Tokyo, Japan; ^2^ Division of Immune Homeostasis, Department of Pathology and Microbiology, Nihon University School of Medicine, Tokyo, Japan; ^3^ Gabrichevsky Research Institute of Epidemiology and Microbiology, Moscow, Russia; ^4^ Microbiology and Immunology, Nihon University, School of Dentistry at Matsudo, Chiba, Japan; ^5^ Department of Food Science, Jumonji University, Saitama, Japan

**Keywords:** female reproductive tract, microbiota, vagina, uterus, ovary, gut, brain, oral cavity

## Abstract

The female reproductive tract (FRT) and remote/versatile organs in the body share bidirectional communication. In this review, we discuss the framework of the “FRT-organ axes.” Each axis, namely, the vagina-gut axis, uterus-gut axis, ovary-gut axis, vagina-bladder axis, vagina-oral axis, uterus-oral axis, vagina-brain axis, uterus-brain axis, and vagina-joint axis, is comprehensively discussed separately. Each axis could be involved in the pathogenesis of not only gynecological diseases but also diseases occurring apart from the FRT. Although the microbiota is clearly a key player in the FRT-organ axes, more quantitative insight into the homeostasis of the microbiota could be provided by host function measurements rather than current microbe-centric approaches. Therefore, investigation of the FRT-organ axes would provide us with a multicentric approach, including immune, neural, endocrine, and metabolic aspects, for understanding the homeostatic mechanism of women’s bodies. The framework of the FRT-organ axes could also provide insights into finding new therapeutic approaches to maintain women’s health.

## Introduction

The gastrointestinal (GI) tract and remote organs in the body share bidirectional communication *via* immune, neural, endocrine, or metabolic links called the “gut-organ axis” (1). Organs such as the brain, skin, bone, liver, heart, and kidney have been known to communicate multidirectionally with the GI tract, and the microbiota appears to be the key player within these axes. Metabolites produced by the microbiota contribute to the metabolic phenotype of the host and alter the risk of disease (2–4).

The female reproductive tract (FRT) is composed of the lower FRT (vulva, vagina, and ectocervix) and the upper FRT (endocervix, uterus, fallopian tubes, and ovaries). Accumulating evidence has suggested the existence of a vagina-organ axis, such as the vagina-gut axis and vagina-bladder axis (5, 6). Since the vagina is exposed to various pathogens, the physiological condition and microbiota of the lower reproductive tract are crucial for women’s health (7–11). In addition, knowledge of the vagina-organ axis may promote further understanding of not only vaginal health but also the health of other organs apart from the vagina. For example, a nationwide study of American women revealed that dysbiotic conditions in the vagina were significantly associated with periodontitis (12). In addition to the vagina-organ axis, the uterus-organ axis could also be involved in a variety of physiological roles. Recent evidence suggests that the endometrial microbiota appears to be involved in fertility and uterine-related diseases (13, 14). Therefore, modulation of the endometrial microbiota *via* the uterus-gut axis may play a critical role in fertility (15). In addition, a study showed that short-chain fatty acids (SCFAs) produced by the maternal gut microbiota influenced the metabolic programming of offspring during pregnancy (16), indicating the importance of the uterus-gut axis not only in fertility but also in the development of offspring. Furthermore, recent studies have indicated an interaction between the uterus and brain (17). Overall, the physiological condition of the FRT could be important for the systemic health of women.

In this review, we discuss the framework of the “FRT-organ axes”, i.e., bidirectional communication between the FRT and remote/versatile organs. We provide an overview of each axis, namely, the vagina-gut axis, uterus-gut axis, ovary-gut axis, vagina-bladder axis, vagina-oral axis, uterus-oral axis, vagina-brain axis, uterus-brain axis, and vagina-joint axis. Innate and adaptive immunity regulate the composition of the microbiota and vice versa (18–22). The production of antimicrobial peptides and proteins (AMPs) and mucus is directly triggered *via* the activation of pattern-recognition receptors, such as Toll-like receptors and NOD-like receptors, by the microbiota, while AMPs and mucus can affect the composition of the microbiota. Immunization with microbes also induces immunoglobulins (Igs), which regulate the composition of the microbiota. In addition to such an immunological route, the neuronal route, the endocrine route, and the metabolic route connect the FRT and distal organs (17). We also discuss potential therapeutic opportunities to restore the microbiota, as a variety of treatments have been tried as approaches to women’s health, and vaginal probiotics seem somewhat promising (17, 18). Finally, future directions in this field are discussed.

## Microbiota in the FRT

Although the surface area of the vaginal mucosa is much smaller than that of the gastrointestinal and respiratory mucosa, the presence of a very large number of bacteria, dominated by a single or a few species of bacteria, is characteristic. Investigations using culture-independent techniques have revealed that the human vaginal bacterial community is dominated by *Lactobacillus*. The vaginal microflora is classified into five community state types: *L. crispatus* dominant (CST I), *L. gasseri* dominant (CST II), *L. iners* dominant (CST III), *L. jensenii *dominant (CST V), and CST IV, which contains anaerobic bacteria, such as *Gardnerella vaginalis* and *Prevotella bivia* (bacterial vaginosis (BV)-associated bacteria), and a relatively low population of *Lactobacillus* (23, 24). Many factors, such as age, ethnicity, menstrual cycle, lifestyle, immune system, infection, and probiotics, can affect the composition of the vaginal microbiota [for review, see (25–27)].

In contrast to the vaginal microbiota, the composition of the normal endometrial microbiota is still under debate (28, 29). In particular, whether *Lactobacillus* dominates in the endometrium needs to be clarified (14, 30, 31). A recent study revealed that the directionality of bacterial translocation was from the vagina to the uterus (32). Compared to that in the vagina, the total amount of bacteria decreases by more than three orders of magnitude in the endometrium (median fold change in Cq value was 1.75), which was previously thought to be sterile (31, 33). Therefore, possible contamination of vaginal microbiota during sampling may disturb the results of the investigation of endometrial microbiota, especially in the detection of dominant species. Although the uterine endometrium is even smaller and contains very few bacteria in absolute terms, it appears to play an important biological role in nurturing fetuses. For example, *L. crispatus* significantly promoted invasion of the trophoblast cell line (34). Low amounts of *Fusobacterium nucleatum* also promoted the invasion of trophoblast cells and induced the secretion of mediators for pregnancy establishment (35). Further studies in this field are strongly needed to establish adequate reproductive medicine. Although the placenta has been reported to harbor a microbiota similar to that of the oral cavity (36), some studies have questioned the existence of the placental microbiota, especially in pregnant women at term (37). A recent systematic review using structured quality bias assessment concluded that the existence of a low biomass placental microbiota in healthy pregnancies cannot be disproved by current evidence (38). Compared with the vagina, the fallopian tube has microbiota with higher diversity (*Acinetobacter*, *Burkholderia*, *Comamonas*, *Coprococcus*, *Corynebacterium*, *Enterococcus*, *Lactobacillus*, *Prevotella*, *Propionibacterium*, *Pseudomonas*, *Staphylococcus*) (33, 39, 40). However, little is known about the roles of the microbiota in the fallopian tube.

In addition to the bacteriome, the virome, mycobiome, archaeome and candidate phyla radiation also consist of the microbiome of FRT (30, 41–43). *Trichomonas vaginalis*, the causative agent of trichomoniasis, is also a well-known protist parasite in the vagina (44). The virome in FRT, especially bacteriophages, appears to be a potential player in the establishment and maintenance of vaginal dysbiosis (45). However, little is known about the role of these biomes in FRT-organ axes.

## Anatomical overview of the FRT-organ axes

**Figure 1:**
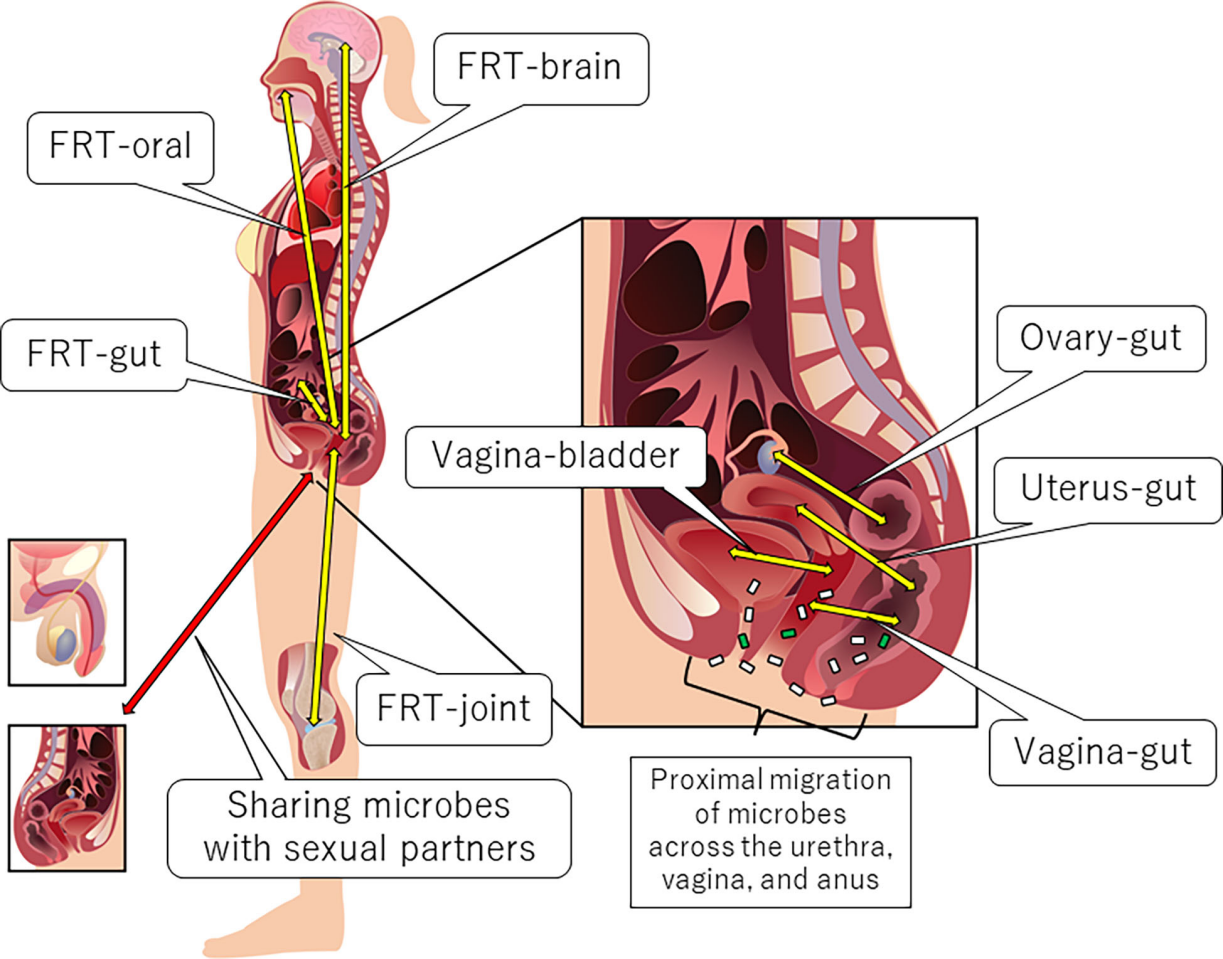
Anatomical overview of the FRT-organ axes. Each axis can be characterized by proximal or distal interactions. Trafficking of bacterial species is a unique aspect of proximal axes compared to other distal axes. However, distal bacterial translocation might occur systemically *via* the bloodstream, especially in pathological conditions accompanied by the dysregulation of epithelial barrier function.

Anatomically proximal and distal interactions comprise the FRT-organ axes (5) (**Figure 1**). The openings of the urethra and vagina are in close proximity to each other. Therefore, some microbial species can be transient members of the microbiota between the bladder and the vagina (vagina-bladder axis). Although the vagina-gut axis is characterized by distal interactions (5), the rectum is relatively close to the vagina. Bacteria can also directly migrate from the anus to the vagina and urethra (5, 46). In addition to bacteria, fungi such as *Candida albicans* may migrate between the anus and the vagina (47). Furthermore, recent studies indicate that the interaction between the microbiota and immunity in the small intestine is different from that in the colon (48, 49), suggesting that we may separate the vagina-gut axis into the vagina-small intestine axis, the vagina-colon axis, and the vagina-rectum axis. In this review, however, we still use the phrase “vagina-gut axis” because few studies have analyzed the microbiota and its effects on immunity in the small intestine. Some studies have also reported distal interactions of the FRT with the ovary, oral cavity, brain, and joints. For example, bacterial translocation could occur from the oral cavity or gut *via* the bloodstream to the uterus (5, 28, 50). Microbial translocation also occurs during sexual intercourse, and some urogenital microorganisms are shared with sexual partners, which is beyond the scope of this review [for review, see (5, 51)]. Below, we describe each axis in detail.

## Vagina-gut axis

Among the FRT-organ axes, the vagina-gut axis is the most studied and discussed. Reducing the risk of BV (dysbiotic condition of the vaginal microbiota) by a healthy diet (52, 53) has enhanced the importance of the interaction between the GI tract and the vagina. One of the oldest descriptions of the term “gut-vagina axis” was referred to by Ravel and Brotman in 2016 (54). Trafficking of bacterial species across the vagina and gut, as mentioned above, is a unique aspect of proximal axes compared to other distal axes. Some *Lactobacillus* strains are found in both the rectum and the vagina (55). In a previous study, 63 bacterial species were identified in the vagina or rectum, 44% of which were found in both organs, and the genotypes of 68% of the species were identical (56). A recent study showed that BV-associated bacteria in the vagina and rectum in pregnant women were individually detected by species-level Spearman correlation coefficient analysis (57). Furthermore, orally administered probiotic strains were also detected in the vagina (58–61). These observations suggest that the rectum could preserve vaginal bacteria (15, 57) and, possibly, vice versa. Importantly, a genomic study of *L. crispatus* and *L. rhamnosus* from a variety of sources, including the human gut and vagina, suggested body site-specific adaptation (62, 63). *L. crispatus* and *L. gasseri* from the reproductive tract and GI tract exhibited source-dependent gene expression and phenotypic characteristics, such as growth rates, stress resistance, adhesion properties, and fermentation profiles, in addition to strain-dependent differences (64). Hence, the preference of *Lactobacillus* for each organ should be considered when discussing the sharing of *Lactobacillus* by both the rectum and the vagina, although further research is needed.

In addition to direct bacterial translocation, indirect interaction between the vagina and gut has also been implicated. Metabolites produced by the microbiota, such as SCFAs, could be considered indirect players of the vagina-gut axis. The roles of SCFAs are prominently different between the vagina and gut [for review, see (15)]. SCFAs in the gut exhibit beneficial functions, such as maintenance of barrier functions, while those in the vagina are associated with dysbiotic and proinflammatory conditions (65). SCFAs can also influence immune cell subsets and their functions (66). As SCFAs produced by the gut microbiota can be transferred to other organs *via* systemic circulation (67), SCFAs may be involved in the vagina-gut axis. SCFAs produced by vaginal microbes are thought to contribute to the development of a dysbiotic environment (65). An *in vitro* study showed that excessive SCFAs can be a potential source of cervicovaginal inflammation (68). Therefore, the circulation of SCFAs from the gut to the vagina might cause vaginal dysbiosis.

**Figure 2:**
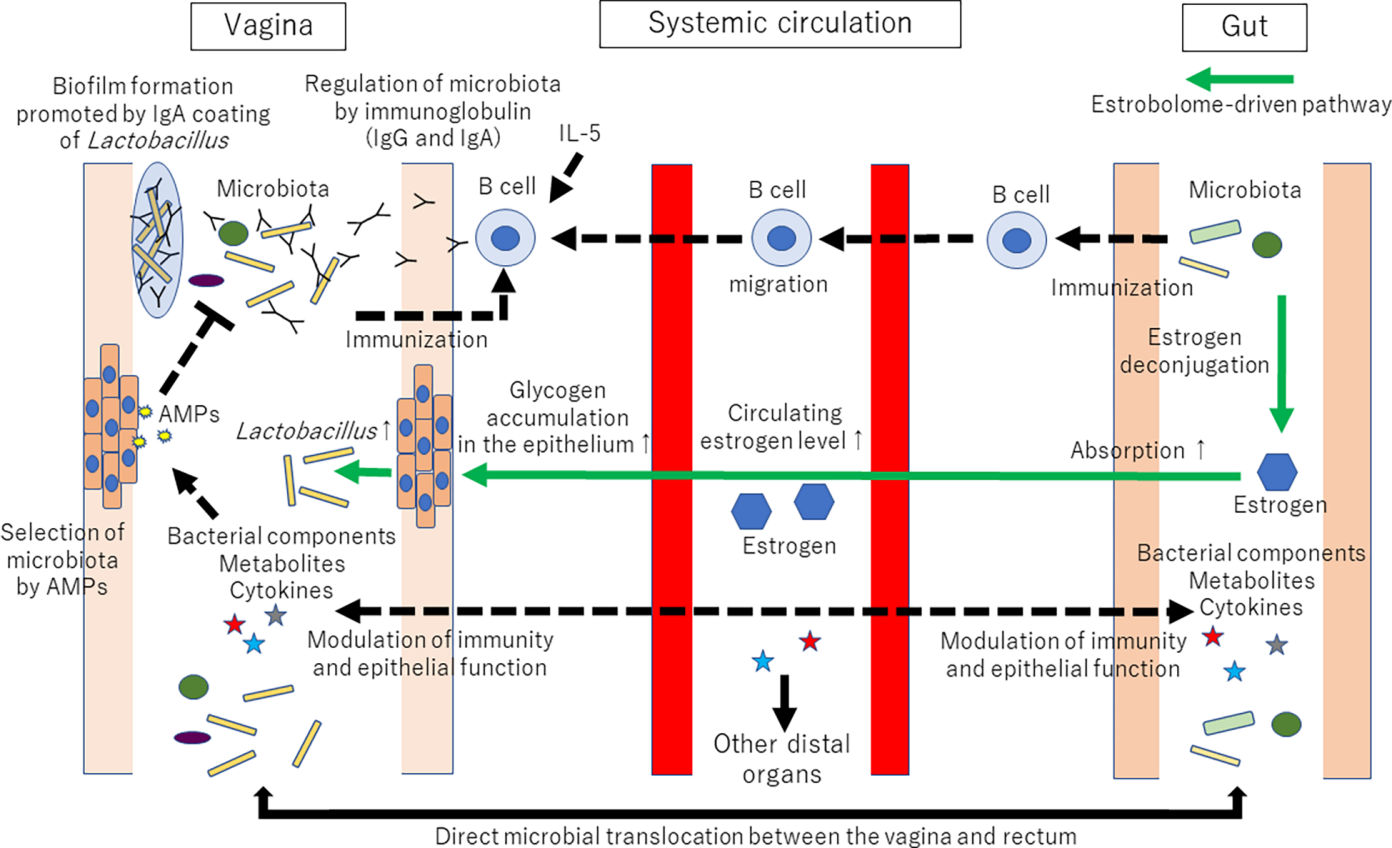
Proposed mechanisms of vaginal microbiota shaping *via* the vagina-gut axis. Many factors, such as age, ethnicity, menstrual cycle, lifestyle, immune system, infection, and probiotics, are known to affect the composition of the vaginal microbiota. However, why the human vaginal microbiota is often dominated by a single or a few strains of *Lactobacillus* is still unknown. Tolerance and promotion of *Lactobacillus* dominance in the vagina might be regulated *via* the vagina-gut axis. Immunization with *Lactobacillus* might occur both in the vagina and the gut. The collection of microorganisms that can metabolize estrogens (coined the estrobolome) can deconjugate hepatically conjugated estrogens in the gastrointestinal tract. Deconjugated estrogen is then reabsorbed to the systemic circulation. Circulating estrogen will affect the distal epithelia of the vagina, resulting in glycogen and mucus production. Then, increased glycogen supports *Lactobacillus* dominance in the vagina because glycogen can serve as an energy source for lactobacilli. AMPs: antimicrobial peptides and proteins. The established pathways are represented by solid lines. The proposed mechanisms are represented by dotted lines. The estrobolome-driven pathway is represented by green lines.

In addition to metabolites, sex hormones contribute to the vagina-gut axis. The vaginal microbial community can be influenced by an estrobolome-mediated mechanism [for review, see (5, 69, 70)]. Briefly, the estrobolome is defined as the collection of microorganisms that can metabolize estrogens. For example, *Bifidobacterium*, *Clostridium*, and *Lactobacillus* are involved in the deconjugation/conjugation of estrogens (71, 72). The estrobolome can deconjugate hepatically conjugated estrogens in the GI tract. Deconjugated estrogen is then reabsorbed to the systemic circulation. Circulating estrogen reaches the distal epithelium of the vagina and alters the physiological characteristics of vaginal epithelial cells, such as glycogen and mucus production. Increased glycogen supports *Lactobacillus* dominance in the vagina, as glycogen can serve as an important energy source for lactobacilli (73, 74). Thus, the amount of estrogen-metabolizing bacteria in the gut microbiota can affect the amount of *Lactobacillus* in the vaginal microbiota. The proposed mechanisms of vaginal microbiota shaping *via* the vagina-gut axis are shown in **Figure 2**.

One of the most important immunological roles of the vagina-gut axis is to shape the components of the vaginal microbiota. Some factors, such as AMPs secreted from epithelial or immune cells from the mucosal site of hosts, regulate the microbiota and vice versa (21, 26). IgA also regulates the composition of the gut microbiota (18–20, 22). IgA is the principal antibody class secreted in mucosal fluid (75, 76). The origin of most IgA in the gut is as a B-cell response to commensal bacteria (77). However, unlike other mucosal tissues, the IgA level is lower than the IgG level (78, 79), and IgG instead dominates antigen-specific antibodies in the vagina (80). This difference can indicate a different defensive function between the vagina and other mucosal tissues (81). IgA and IgG appear to be stably associated with cervical mucus, while only IgG appears to be associated with cervicovaginal mucus (82). IgA-Seq (bacterial flow cytometry with 16S rRNA gene sequencing) revealed that lactobacilli were coated with IgA *in vivo* (83). In contrast, most bacteria in the Firmicutes phylum were not coated with IgA (83). Of note, Breedveld and colleagues recently reported that higher levels of bacterial IgA coating were observed when the vaginal microbiota was dominated by *L. crispatus* compared with non-*L. crispatus*-dominated microbiota (84). The bacterial IgA coating might modulate biogeography, metabolic processes, proliferation, and survival in mucosal tissue by modifying bacterial gene expression (85, 86). IgA coating is proposed to promote adhesion to the mucus layer and facilitate the formation of bacterial biofilms on the mucosal surface, which is important for bacterial colonization in the gut (87). Therefore, similar to its regulatory function in the GI tract, IgA could play a role in maintaining the *L. crispatus*-dominated microbiota (84). A previous study showed that genetic variants of IL-5 were associated with the abundance of *Prevotella* spp. in the vaginal microbiota (88). As IL-5 is implicated in IgA responses (89), host genetics could affect the vaginal microbiota *via* immunological regulation. Furthermore, competition among *Lactobacillus* species of the vaginal microbiota appears to be less likely to occur (90, 91). These studies might support the importance of host factors, rather than bacterial competition of lactobacilli, in shaping the *Lactobacillus*-dominant vaginal microbiota (91).

Furthermore, questions arise about the immunization of vaginal *Lactobacillus*, specifically when and where immunoglobulin binding to *Lactobacillus* could be induced. The components of the vaginal microbiota dramatically change throughout a woman’s life (15, 69). Similar to a mother’s vaginal microbiota, the meconium of newborns delivered vaginally contains microbes such as *Lactobacillus* and *Prevotella* (92), suggesting that one of the sources of the infant’s gut microbiota would be the mother’s vaginal microbiota. Interestingly, *L. crispatus* from the mother’s vagina might play an important role in establishing the mucosal barrier function of the infant gut (93). In contrast, after birth and during childhood, the vaginal microbiota does not resemble that of the mother and contains streptococci, enterococci, and anaerobes with high microbial diversity, and *Lactobacillus* is deficient (94). Therefore, the immunization of *Lactobacillus*, which will be the dominant vaginal species, is less likely to occur in the vagina before puberty. Instead, as the rectum could preserve vaginal bacteria (15), immunization of vaginal *Lactobacillus* after puberty might occur in the gut before puberty. After puberty, with an increase in circulating estrogen levels and accumulation of glycogen in the thick vaginal epithelium as an energy source for *Lactobacillus* (73, 74), the vaginal microbiota is dominated by one or a few *Lactobacillus* species. After menopause, the estrogen level decreases, resulting in a reduction in *Lactobacillus* abundance.

After puberty, immunization with *Lactobacillus* might also occur in the vagina, in addition to the gut. Although the importance of the local immune structures in the vagina is still under investigation (95), the vaginal microbiota can stimulate the mucosa continuously. Therefore, B cells that respond to vaginal commensal organisms might be recruited to the vaginal mucosa, and IgG and IgA secreted from these B cells might have a role in shaping the microbiota in the vagina. Furthermore, *L. crispatus* might have immunomodulatory capabilities mediated by the S-layer protein, which comprises the outer surface layer of the bacteria (96). In addition, S-layer protein was reported to be a mediator of *Lactobacillus* adhesion to epithelial cells (93, 96, 97).

Although the mechanism of immunizing with *Lactobacillus* as a component of the vaginal microbiota is uncertain, investigations of genital mucosal antibodies after vaginal challenge with pathogens have provided some insights. In mice, after vaginal infection immunization with herpes simplex virus 2 (HSV-2), circulating antibodies rarely entered the vaginal lumen, and this primary infection did not recruit plasma cells in the vaginal mucosa (98). However, after secondary vaginal challenge with HSV-2, induction of CXCL9 and CXCL10 by IFN-γ from tissue-resident memory T cells in the vagina recruited circulating memory B cells to the vaginal mucosa in a CXCR-3-dependent manner, and the B cells secreted virus-specific IgG and IgA into the vaginal lumen (98). In macaques, two vaginal challenges and three oral Simian immunodeficiency virus challenges induced virus-specific IgA and IgG antibodies in the vaginal fluid and serum (99). This vaginal IgA had a J chain and a secretory component, indicating that it was of secretory origin (99). In humans, vaginal vaccination with the cholera toxin B subunit increased the concentrations of IgG and IgA in cervical secretions, while nasal vaccination showed less induction of IgG and IgA (100). In contrast, more induction in vaginal IgA secretions was observed when the antigen was administered nasally (100). To date, however, a mechanism for establishing a vaginal microbiota has not been developed.

The vagina-gut axis appears to be involved in disease development. The presence of *G. vaginalis* in the gut microbiota significantly increased the odds of irritable bowel syndrome (IBS) (101). *Gardnerella* was significantly more enriched in the gut microbiota of patients with ulcerative colitis than in patients with Crohn’s disease (102). These results may represent translocation or contamination of bacteria from the vagina before or during sampling. However, a significant link between inflammatory bowel disease (IBD) and the urogenital biofilm of *G. vaginalis* in urine specimens suggested dysfunction of the vaginal epithelial barrier (103). Recent studies have also indicated the association of vaginal dysbiosis and IBD in pregnant women. Pregnant women with IBD had a risk for BV and *Candida* colonization (104). Although the causal relationship was unknown, the prevalence of *Mycoplasma* and/or *Ureaplasma* was also significantly higher in pregnant women with IBD than in pregnant women without IBD (105).

The link between BV and IBD might also be explained by lipopolysaccharide (LPS), a component of the membrane of gram-negative bacteria. As a consequence of the downregulation of intestinal barrier integrity induced by gut dysbiosis accompanied by IBD, LPS can translocate into the systemic circulation (106, 107), causing acute or chronic inflammation at distal sites (108). Although there is no evidence showing that vaginal dysbiosis could increase circulating LPS, LPS from the gut might cause/worsen inflammation at the vagina.

Furthermore, infection of *G. vaginalis* in murine vaginas increased the concentration of TNF-α and myeloperoxidase activity and decreased the concentration of IL-10 in colon tissue (109). This infection also decreased the Bacteroidetes composition and increased the Proteobacteria composition in the gut microbiota (109). Overall, vaginal dysbiosis might be a trigger/aggravating factor of these bowel diseases and perhaps vice versa.

An association between the gut microbiota and cervical cancer has also been suggested. Altered diversity and composition of the gut microbiota of patients with cervical cancer have been reported (110). SCFAs inhibited the proliferation of HeLa cells (human cervical cancer cell line) *via* the downregulation of free fatty acid receptor 2 expression (111). Therefore, such metabolites produced by the gut microbiota could indirectly affect the progression of cervical cancer (72). Oral lactic acid bacteria (LAB)-based mucosal human papillomavirus (HPV) vaccines may influence the course of cervical cancer (persistent infection with HPV is associated with oncogenesis of cervical cancer) through the flow of immune signals from the gut to the vagina (112). In addition, a meta-analysis showed a causal link between cervical cancer and vaginal dysbiosis (113). Therefore, another indirect link between cervical cancer and the gut microbiota can be postulated if vaginal dysbiosis is induced *via* the vagina-gut axis.

To consider the mechanism of the vagina-gut axis, one suggestive example is *Helicobacter* infection in the stomach. *H. pylori* is highly virulent in the stomach (114), and some studies have shown an inverse correlation between *H. pylori* infection and IBD onset, indicating that *H. pylori* could induce systematic immune tolerance and the suppression of the inflammatory response (115). In contrast, recurrent BV can be cured after antibiotic therapy for *H. pylori* (116). Although antibiotic therapy for *H. pylori* can directly change the vaginal flora, *H. pylori*-induced systematic immune tolerance might promote colonization of pathogens in the vagina. Therefore, these studies might represent a double-edged sword of gastric *H. pylori* infection to modulate systemic mucosal immune modulation, including the vagina-gut axis.

## Uterus-gut axis

The uterus has unique immune regulation (117, 118). However, how uterine immunity and its microbiota interact with each other requires further investigation. The hematogenous spread of bacteria from the gut to the uterus has been reported (28). Recently, an association between the gut microbiota and preterm birth has been highlighted. Reductions in *Bifidobacterium*, *Clostridium*, and *Bacteroides* have been observed in the gut microbiota of patients with preterm birth (119, 120). As these bacteria have many anti-inflammatory properties, such as inhibition of LPS-induced NF-κB activation, IL-8 and COX-2 production, and induction and activation of IL-10-secreting regulatory T cells, a reduction in these bacteria could lead to increased susceptibility to inflammation-induced preterm birth (46).

The estrobolome is involved in not only the vagina-gut axis but also the uterus-gut axis. Endometrial hyperplasia and cancer are estrogen-dependent (29). Therefore, the estrobolome in the gut can influence the development of these estrogen-dependent diseases (5, 17, 70, 72).

Active IBD can impair fertility, probably *via* multifactorial mechanisms, including pelvic inflammation of the ovaries and fallopian tube (121). The change in endometrial microbiota accompanied by IBD remains unknown, although predominant taxa in the endometrial microbiota for different gynecological disorders have gradually become clear (14). LPS from the gut might impair fertility and preterm birth by inducing apoptosis of embryonic cells, delaying stromal cell proliferation, and modulating hormones (122).

Interestingly, SCFAs from the maternal gut microbiota affected the metabolic phenotype of offspring in mice during pregnancy (16). Therefore, the uterus-gut axis can play roles not only in fertility but also in the development of offspring.

## Ovary-gut axis

Ovarian functions, including ovulation and lutein body formation, are precisely controlled by pituitary hormones but are also affected by intestinal functions, including the microbiota. For example, IBD can be a risk factor for ovarian dysfunction, including premature ovarian failure (123). Furthermore, patients with polycystic ovary syndrome (PCOS), which is often accompanied by hyperandrogenism, showed reduced diversity and different phylogenetic compositions of the gut microbiota (124, 125). *Bacteroidaceae*, *Bacteroides*, *Coprococcus*, *Escherichia/Shigella*, *Faecalibacterium prausnitzii*, *Lactobacillus*, *Parabacteroides*, and *Prevotella* were identified as the most common bacterial alterations in PCOS patients (126). Interestingly, SCFAs restored ovarian function in a PCOS rat model (127). Insulin resistance with abnormal SCFA metabolism by the gut microbiota might be associated with PCOS (128). Therefore, regulation of SCFA by the gastrointestinal/vaginal microbiota could be involved in the pathogenesis of PCOS. In addition, as IL-22 produced by intestinal group 3 innate lymphoid cells (ILC3) reversed insulin resistance (129), interactions among the gut microbiota, bile acid, and IL-22 appear to have roles in the pathophysiology of PCOS (130). A chronic inflammatory state and disrupted gut mucosal integrity caused by gut dysbiosis are other inducible candidates for PCOS (131). A previous study also showed that an increased prevalence of BV was found in patients with PCOS (132). Hence, the vagina-gut axis in addition to the ovary-gut axis might be important in PCOS patients (131).

In addition to PCOS, the gut microbiota can modulate ovarian cancer. The presence of sex hormone receptors in many putative tissues of origin for ovarian cancer suggests potential roles for sex hormones in the origin and promotion of ovarian cancer (133). Therefore, the estrobolome in the gut might have an important role in the induction of estrogen-dependent ovarian cancer (72).

In terms of reproduction, LPS from the gut might affect not only the uterus but also the ovary. LPS can activate macrophages in the ovaries, which produce cytotoxic proinflammatory cytokines (134, 135). These cytokines could exert autocrine/paracrine effects causing regression of the ovary (122).

## Vagina-bladder axis

Common genera of the urinary microbiota are *Citrobacter*, *Escherichia*, *Enterococcus*, *Prevotella*, and *Streptococcus* (136, 137). *Corynebacterium* and *Streptococcus* are reported to be more abundant in men, and *Lactobacillus* is more abundant in women (137). The urinary microbiota shares 62.5% of species with the gut microbiota and 32% with the vaginal microbiota (137). As urine samples showed substantial concordance to paired mid-vaginal samples regarding bacterial composition, the genitourinary microbiota could be a good indicator for the overall composition of the vaginal microbiota (138).

A typical starting point of a urinary tract infection (UTI) is periurethral contamination by a uropathogen residing in the gut (139). Then, the uropathogen colonizes the urethra and subsequently migrates to the bladder. Therefore, dysbiotic states with pathogenic microbes in the gut would increase the risk of UTI (140). Similar to this gut-bladder axis, vaginal dysbiosis may prompt a UTI *via* the vagina-bladder axis as a reservoir for uropathogens such as *E. coli* (6). Although *G. vaginalis* is a rare cause of symptomatic UTI, an *in vitro* study showed that *G. vaginalis* altered bladder gene expression and increased susceptibility to subsequent UTI caused by uropathogenic *E. coli* (141). Compared to women without BV, those with BV have a significantly increased risk of UTI, with an odds ratio of 13.75 (142), and a clinical trial showed that the efficacy of intravaginally administered *L. crispatus* probiotics reduced the recurrence of UTI (143). Furthermore, as *G. vaginalis* is often detected in the urinary microbiota (141), the urine microbiota may prompt BV *via* the vagina-bladder axis as a reservoir for *G. vaginalis*.

## FRT-oral axes

Following the GI microbiota, the oral cavity harbors the second most diverse and largely populated microbiota in the body (144). Different sites and conditions in the oral cavity have different microbial compositions (145–147). Marginal gingival biofilms contain *Corynebacterium*, *Streptococcus*, *Neisseria*, *Fusobacterium*, *Leptotrichia*, *Porphyromonas* and *Haemophilus*, while dorsal tongue biofilms contain *Rothia*, *Veillonella*, *Actinomyces*, *Streptococcus*, and *Neisseria* (148). Cooperation of the oral microbiota and the host reflects the information and status of immunity and metabolism *via* a two-way axis along the oral cavity and systemic organs (149). Below, we discuss the vagina-oral axis and the uterus-oral axis.

## Vagina-oral axis

Evidence indicates the existence of the vagina-oral axis. There is a significant association between periodontitis and BV (150). Women with BV have a higher risk of gingivitis (151). An association with a significant diversification of salivary microbiota and higher counts of *Prevotella intermedia* in the subgingival gingival microbiota was reported in women with BV compared with women without BV (152). Sexual risk behaviors such as oral sex could translocate pathogens between the oral cavity and the vagina and would be one mechanism of the vagina-oral axis (150). Furthermore, a recent study showed an association between periodontitis and higher systemic markers of inflammation in women with BV, indicating systemic inflammation as the underlying mechanism (12). The coexistence of BV and periodontitis in smokers (12) is also good evidence of the vagina-oral axis. The association was previously thought to be due to women who smoke being more sexually active than nonsmokers, but more recently, it has been suggested that several chemical factors in cigarettes, together with common abnormalities in mucosal immunity (153), might kill oral and vaginal commensal bacteria directly or *via* bacteriophages (154, 155).

## Uterus-oral axis

The relationship between labor onset and the oral cavity raises interesting questions. In general, dental caries and periodontal disease are associated with preterm labor (156). Although the existence of a normal placental microbiota is under debate, as mentioned above (37, 38), some studies have suggested that inflammatory conditions of the oral cavity could cause bacterial translocation from the oral cavity *via* the bloodstream to the uterus (157–159). DNA of periodontal disease-related pathogens, such as *Porphyromonas gingivalis*, *Fusobacterium nucleatum*, *Prevotella intermedia*, and *Treponema denticola*, is often detected in the placenta of patients with periodontal disease (160). Such pathogens can cause placental dysfunction (161). Furthermore, the presence of *P. gingivalis* in the umbilical cord is associated with preeclampsia. The genus *Aggregatibacter*, which includes some periodontal disease-related bacteria, is significantly enriched in the oral microbiota of women with threatened preterm labor (57). Overall, oral bacteria may reach the uterus in the bloodstream and cause inappropriate labor, either directly or *via* inflammatory cytokines. Therefore, an association between periodontal disease and adverse pregnancy outcomes is suggested (162), although further mechanisms of the uterus-oral axis are still under investigation.

## FRT-brain axes

The gut-brain axis consists of multiple connections, such as bacterial metabolites and products, the vagus nerve, and the immune system [for review, see (163, 164)]. Therefore, similar physiology could be postulated in the FRT-brain axis. Microbial-derived metabolites appear to be modulators of the FRT-brain axis. For example, SCFAs produced by the GI microbiota are speculated to have a key role in the gut-brain axis (67), indicating that the production of SCFAs in the vagina might affect brain function similarly. Although it is still unknown whether an efferent vagal influence on pelvic organs exists, vagal afferent supply of the uterus has been indicated by retrograde and anterograde tracing studies *in vivo* (165). Normal immune function could be impaired or dysregulated by exposure to chronic stress through the hypothalamic−pituitary−adrenal pathway and the sympathetic-adrenal-medullary pathway with associated hormones (166). Some studies have indicated the existence of the vagina-brain and uterus-brain axes, as discussed below. However, the FRT-brain axis needs further investigation.

## Vagina-brain axis

Stress might affect the vaginal microbiota (27). Stress-induced cortisol could inhibit vaginal glycogen deposition, resulting in lower *Lactobacillus* dominance, elevated vaginal pH, and an increase in the proinflammatory response (167). Increased psychosocial stress is associated with greater BV prevalence (168), although few associations between stress and BV have been observed in pregnant women (169). Another study reported no significant associations between the vaginal microbiota and mood, although the sample size was limited (170). SCFAs produced by the gut microbiota may regulate brain function directly or indirectly (67). Therefore, similar functions can be assumed in SCFAs from the vaginal microbiota.

## Uterus-brain axis

Accumulating evidence is providing a clearer understanding of the brain-uterus axis (17). Gonadotropin releasing hormone (GnRH) signals the pituitary gland to secrete luteinizing hormone (LH) and follicle-stimulating hormone (FSH), which consequently increase the production of estrogens from the ovary (171, 172). Adenomyosis and endometriosis are estrogen-dependent gynecological disorders that cause pelvic pain, abnormal uterine bleeding, and infertility (173, 174). GnRH agonists or antagonists are administered to treat these diseases, and improvements in chronic pelvic pain have been reported (175). It is hypothesized that pain sensitivity might increase the secretion of GnRH (17). Therefore, further studies of pelvic pain and GnRH secretion from the pituitary gland would promote our understanding of the uterus-brain axis.

## Vagina-joint axis

Some studies have reported the involvement of the vagina-joint axis in joint diseases. Reactive arthritis and rheumatoid arthritis have been revealed to be associated with BV and *Gardnerella* in the gut microbiota (176–178). Although the mechanisms of interactions between the microbial antigen and the host remain unknown, the cause of reactive arthritis is considered an overstimulated autoimmune response or bacterial antigens that deposit in the joints (179). The antigens that can trigger reactive arthritis are gram-negative aerobic bacteria, and these bacteria invade gastrointestinal/urogenital mucosal sites (179). Therefore, reactive arthritis could be caused by vaginal *G. vaginalis* (gram-negative) infection (180), which would be considered the vagina-joint axis. In contrast, the mechanism by which vaginal dysbiosis may cause rheumatoid arthritis remains a mystery.

## Therapeutic opportunities

**Figure 3:**
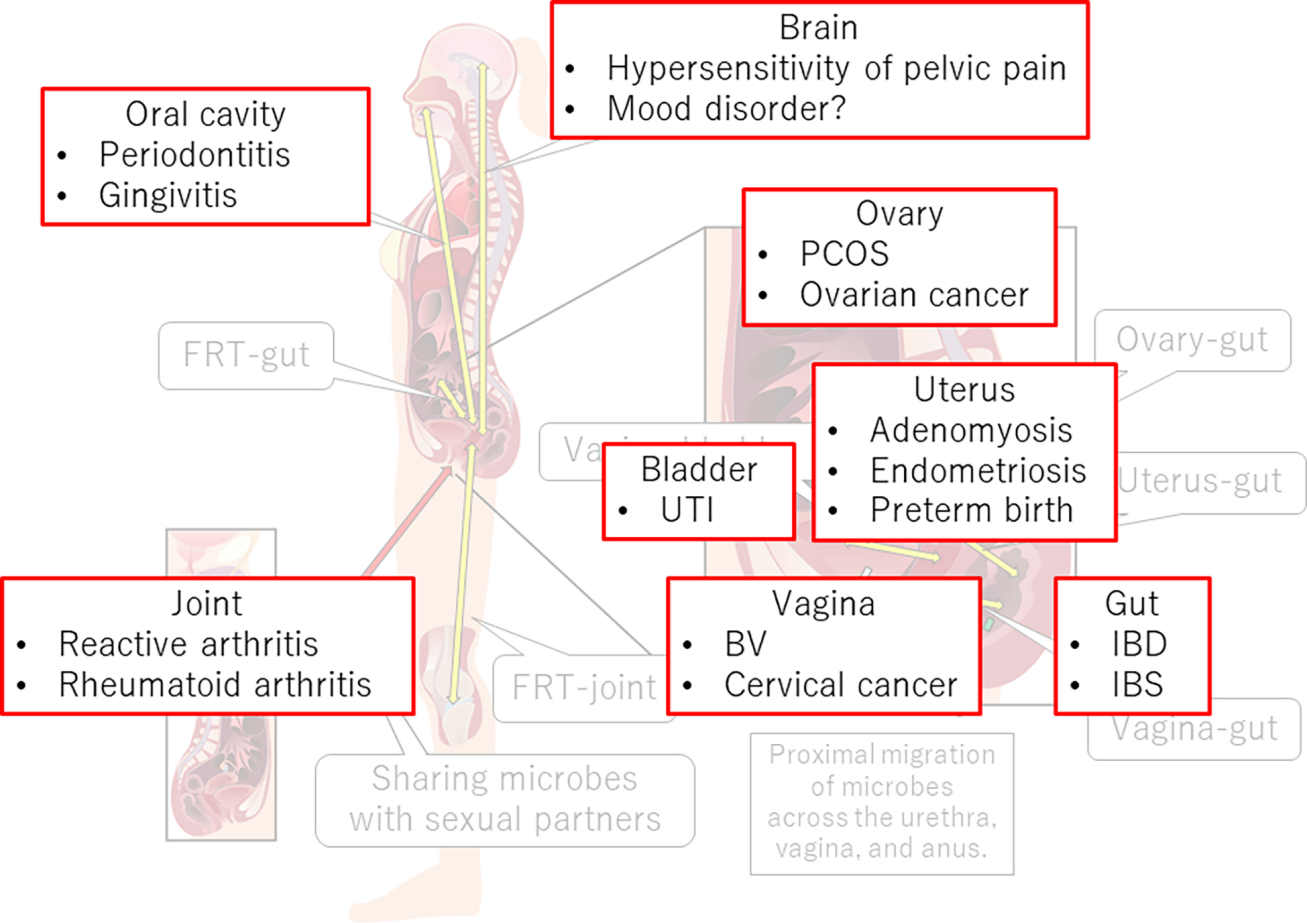
FRT-organ axes and diseases. Dysregulation of immune, neural, endocrine, or metabolic functions in the FRT-organ axes implicates its involvement in the pathogenesis of not only gynecological diseases but also diseases occurring apart from the FRT. PCOS, polycystic ovary syndrome; UTI, urinary tract infection; BV, bacterial vaginosis; IBD, inflammatory bowel disease; IBS, irritable bowel syndrome.

The FRT-organ axes appear to be involved in a variety of diseases (**Figure 3**). Therefore, restoration of dysbiotic conditions is a potential treatment. Therapeutic opportunities for pharmabiotics, including prebiotics, probiotics, synbiotics, and fecal microbiota transplantation, are well acknowledged. Recently, a clinical trial of vaginal microbiota transplantation for BV was conducted (181). However, although *L. crispatus* seems preferable for vaginal application, indigenous vaginal lactobacilli often surpass colonization of the probiotic *L. crispatus* strain from the vaginal source (CTV-05) (182). After 24 weeks, approximately 50% of patients who received this probiotic had not retained the strain (183).

Mutual regulation of mucosal factors and the resident microbiota (76) may explain the insufficient clinical efficacy of pharmabiotics, which are “alien” to the body’s own microbiota and local immunity. The colonization resistance of the mucous membranes prevents exogenous bacteria from colonizing the body. In some cases, the use of commercial probiotics even has a negative effect on the process of microbiota recovery after antibiotic therapy (184). An alternative to commercial probiotics is an approach based on the restoration of the microbiota in the event of dysbiosis by using bacterial strains from a person’s own microbiota. This approach, known as autoprobiotic technology or personalized symbiont therapy (PERST), involves the isolation of individual representatives of the resident beneficial microbiota in pure culture, checking for the absence of genes associated with pathogenicity, accumulating these bacteria by cultivation, and returning them back to the host (185, 186). These autoprobiotic strains easily colonize mucous membranes and do not induce a rejection response from the host’s resident microbiota and immune system. Autoprobiotic treatment is accompanied by recovery of the immune status and distinctive anti-inflammatory effects (185). A prospective randomized study showed that autoprobiotic treatment with lactobacilli was more effective than that with allogeneic strains in restoring the level of lactic acid bacteria in the vagina and reducing the risk of recurrence of BV (187). Thus, instead of the classic application of probiotics, PERST technology based on autologous lactobacilli can be used to cure vaginal dysbiosis-related disorders.

The stability of the microflora partially depends on intra- and interspecies bacterial antagonism (8, 188). Although interspecies antagonism might be less likely to occur among vaginal *Lactobacillus*, as discussed above (90, 91), whole-genome sequencing revealed that the species *L. crispatus* is heterogeneous in the vagina (189). Therefore, to understand this advantage of autoprobiotic strains, in-depth research on intraspecies bacterial antagonism also seems to be needed. Furthermore, regarding FRT organ axes, simultaneous administration of autoprobiotics at different sites might synergistically restore dysbiosis.

## Future direction and conclusion

Dysbiosis can be characterized by an underlying disruption of host functions that regulate the microbiota and microbial metabolism (190). Hence, more quantitative insights into the homeostasis of the microbiota could be provided by host function measurements rather than current microbe-centric approaches (190), although the microbiota is clearly a key player in the FRT-organ axes.

Among the FRT-organ axes, the underlying mechanism shared by the “mucus-mucus” axes (such as the vagina-gut axis and the vagina-oral axis) can be discussed; dysfunction at one mucosal tissue may cause dysfunction at other mucosal tissues. Bacterial direct translocation is important for considering these “mucus-mucus” axes. However, indirect interaction should be noted. It is also well established that mucosal dysregulation and dysbiosis can cause chronic inflammation in distal tissues *via* systemic circulation of inflammatory cytokines (191, 192). As discussed above, systemic inflammation could be the underlying mechanism of the oral-gut axis (12). Dysregulated intercellular junction proteins and barrier properties allow bacterial components (e.g., LPS), bacterial metabolites, and cytokines to enter the systemic circulation, resulting in the dysregulation of other tissues. Overall, systemic modulation of the immune system can be the underlying mechanism of the interactions of mucosal tissues in the FRT-organ axes.

Controversial roles of SCFAs in the development of cervical cancer also remain to be elucidated. As discussed above, SCFAs can cause vaginal dysbiotic and inflammatory conditions (65, 68). A meta-analysis supports a causal link between cervical cancer and vaginal dysbiosis (113). However, SCFAs also have antitumor effects (72, 111). Butyrate can trigger cascades of responses that not only lead to malignancy but also inhibit it (193). Therefore, determining the role of SCFAs in cervical cancer development or prevention is a future need.

In addition, although each axis is discussed separately for a comprehensive presentation in this review, the axes can interact with each other, such as the vagina-uterus-gut axes. Therefore, integration of each axis is required for further understanding of the FRT-organ axes. Investigations of not only the bacteriome but also the virome, mycobiome, archaeome and candidate phyla radiation in FRT are still needed to consider the FRT organ axes.

Furthermore, in the current era of diversity, we should also consider the preferable composition of the microbiota for transgender people (7). For transgender women, the microbiota of the penile skin-lined neovagina is substantially similar to that in women with BV with a very limited number of lactobacilli (194). For transgender men prescribed testosterone, the vaginal microbiota is less likely to have *Lactobacillus* (195). Long-term administration of high-dose testosterone disrupts the normal architecture of the vaginal epithelium and reduces glycogen deposition (196), which could result in a reduction in vaginal *Lactobacillus*. Regarding vaginal microbiota modulation, the neovaginal microflora of transgender women was significantly enriched with lactobacilli following an orally administered mixture of lactobacilli (197). A positive association was also found between intravaginal estrogen administration and the presence of *Lactobacillus* in transgender men (195). However, several key gaps in this field remain in the literature, such as the systemic effect of local estrogen therapy in transgender men (198). Furthermore, the role of the FRT-organ axes in transgender people is completely unknown.

In addition to the investigation of the FRT-organ axis, a more effective approach to cure the dysbiotic condition in FRT is needed. One promising approach in the future may be IgA-coated probiotics. IgA facilitates *Bacteroides fragilis* colonization of the murine gut (18). IgA-coated *L. jensenii*, not IgA-free lactic acid bacterial strains, from the fecal microbiota of a healthy woman significantly inhibits dyslipidemia and gut barrier damage in high-fat diet-fed mice (199). As mentioned above, IgA-coated bacteria are observed more frequently in vaginal microbiota with *L. crispatus* dominance compared with that with non-*L. crispatus*-dominant microbiota (84). Therefore, IgA-coated *L. crispatus* as a probiotic might colonize the vagina longer than current probiotic strains. However, to establish this approach, further age-range studies to investigate IgA-coated bacteria in the vagina are needed.

In conclusion, investigation of the FRT-organ axes would provide a multicentric approach including immune, neural, endocrine, and metabolic aspects for understanding the homeostatic mechanism of women’s bodies. The framework of the FRT-organ axes could also provide a cue to find new therapeutic approaches to maintain women’s health.

## Author contributions

The manuscript was conceptualized by KT and VM. KT, VM and SH wrote the manuscript. RK, SK-A, NT and SH supervised the project. All authors contributed to the article and approved the submitted version.
